# Predictive Factors Affecting the Short Term and Long Term Exodrift in Patients with Intermittent Exotropia after Bilateral Rectus Muscle Recession and Its Effect on Surgical Outcome

**DOI:** 10.1155/2014/482093

**Published:** 2014-07-02

**Authors:** Jason C. S. Yam, Gabriela S. L. Chong, Patrick K. W. Wu, Ursula S. F. Wong, Clement W. N. Chan, Simon T. C. Ko

**Affiliations:** ^1^Department of Ophthalmology and Visual Sciences, The Chinese University of Hong Kong, Hong Kong; ^2^Department of Ophthalmology, Tung Wah Eastern Hospital, Hong Kong

## Abstract

*Purpose*. To determine the predictive factors that affect short term and long term postoperative drift in intermittent exotropia after bilateral lateral rectus recession and to evaluate its effect on surgical outcome. *Methods*. Retrospective review of 203 patients with diagnosis of intermittent exotropia, who had surgical corrections with more than 3 years of followup. Different preoperative parameters were obtained and evaluated using Pearson's correlation analysis. *Results*. The proportion of exodrift increased from 62% at 6 weeks to 84% at 3 years postoperatively. The postoperative drift was 4.3 ± 8.1 PD at 6 weeks, 5.8 ± 8.4 PD at 6 months, 7.2 ± 8.3 PD at 1 year, 7.4 ± 8.4 PD at 2 years, and 7.7 ± 8.5 PD at 3 years. Preoperative deviation and initial overcorrection were significant factors affecting the postoperative drift at 3 years (*r* = 0.177, *P* = 0.011, *r* = −0.349, and *P* < 0.001, resp.). *Conclusions*. Postoperative exodrift along three years occurs in a majority of patients after bilateral lateral rectus recession for intermittent exotropia. The long term surgical success is significantly affected by this postoperative exodrift. A larger preoperative deviation and a larger initial overcorrection are associated with a larger early and late postoperative exodrift.

## 1. Introduction

Intermittent exotropia is the most common form of childhood exotropia [1]. Some patients progress into constant exodeviation, while others remain stable or improve; however, the natural history of this disorder remains obscure [2–4]. Patients with intermittent exotropia tend to develop an exotropic drift following surgical correction over time [5]. Because of this exotropic drift, many authors suggested an initial overcorrection for intermittent exotropia may be required for better long term motor alignment. Raab and Parks [6] advised 10 to 20 prism diopters (PD) of overcorrection and Scott et al. [7] advised a 4 to 14 PD of overcorrection. However, other authors suggested that initial overcorrection cannot inhibit the long term recurrence [8].

Therefore knowledge of factors affecting the exodrift and how this exodrift affects surgical outcome is important for surgical planning and patient counseling. The purpose of this study is to determine the predictive factors that affect the short term and long term exodrift in intermittent exotropia after bilateral lateral rectus recession and to evaluate its effect on surgical outcome.

## 2. Patients and Methods

The study protocol followed the principles of the Declaration of Helsinki and was approved by our institutional review board. The clinical records of patients diagnosed with intermittent exotropia and who underwent bilateral rectus muscle recession surgery at Tung Wah Eastern Hospital and Pamela Youde Nethersole Eastern Hospital from 1993 to 2012 were reviewed. Intermittent exotropia was generally defined as a divergent deviation intermittently controlled by fusional mechanisms and intermittently breaking down into a manifest exotropia and onset after one year of age. Exclusion criteria included (1) a convergence insufficiency-type intermittent exotropia (near angle more than 10 D greater than distance angle), (2) horizontal muscle operation procedures other than bilateral lateral rectus recession surgery, (3) patients who underwent intraoperative or postoperative adjustable suture technique, (4) exotropia caused by previous eye muscle surgery, (5) organic ocular or orbital pathology that would reduce vision, (6) patients with developmental delay or neurologic problems, and (7) postoperative followup of less than 3 years. Patients with ocular conditions most likely associated with the strabismus (amblyopia, latent nystagmus, oblique muscle overaction, dissociated vertical deviation, A pattern, or V pattern) were not excluded. Patients with additional oblique muscle operations were not excluded.

The following information was recorded when available: age at onset and at diagnosis, sex, type of intermittent exotropia, near-distance disparity, intermittent versus constant phase before surgery, age at surgery, follow-up period, preoperative refraction, preoperative and postoperative angles of strabismus, associated ocular motility disturbance, binocular sensory status, and associated amblyopia. Age of onset was defined as the age at which a parent or relative first observed ocular misalignment and recalled values were checked using old patients' photographs. The follow-up period was defined as the period between the last visit and the date of the primary operation. The preoperative refraction was defined as the mean spherical equivalent of both eyes. Amblyopia was defined as best-corrected visual acuity of 6/12 or less in one or both eyes, or a bilateral difference of at least two best-corrected visual acuity lines.

All patients underwent complete ophthalmologic and orthoptic examinations before operation. The angle of deviation was measured at 6 m and 1/3 m using alternate prism cover test. Krimsky test was used for uncooperative patients. All deviations recorded were determined using appropriate spectacle correction. For binocular vision assessment, Worth 4-dot and Titmus stereotesting results were recorded. Surgery was recommended if there was deterioration in the frequency or magnitude of exotropia or if tropia was present more than 50% of the time as determined either by examination or by ophthalmic history. All patients had bilateral lateral rectus muscle recession. Some of them had additional bilateral inferior oblique recession for the correction of the V-pattern. Surgery was performed after exodeviation has become stable (stabilization of angle and degree of control) over 2 or 3 consecutive visits. All operations were performed by 1 of 2 surgeons (STCK or PKWW). Surgical dosages were applied using standard tables. Initial postoperative deviation at week 1 (the earliest measured deviation within first week postoperatively) of surgery was recorded for each patient. Postoperative drift was then calculated for each patient at different time points: 6 weeks (4–8 weeks), 6 months (5–7 months), 1 year (11–13 months), 2 years (23–25 months), and 3 years (35–37 months). Postoperative drift was defined as the difference between the angle of deviation at the particular follow-up period and the angle of deviation at week 1. Distance angle of deviation was used in the calculation of postoperative drift.

### 2.1. Statistical Analysis

All analyses were performed with statistical software (StatLab, SPSS for Windows, version 16.0; SPSS, Inc., Chicago, IL). Pearson correlation analyses were used to assess the effect of each preoperative parameter on the postoperative drift at 6 weeks and 3 years postoperatively. All continuous values were presented as mean ± standard deviation. Difference of exodrift among different types of intermittent exotropia was evaluated using Kruskal Wallis test. Difference of exodrift between initial overcorrected group versus initial undercorrected group, between group with stereopsis versus group without stereopsis, and between success group versus failure group was evaluated using *t*-test. Statistical significance was indicated by a *P* value less than 0.05.

## 3. Results

We identified 203 patients with intermittent exotropia who had operations done from 1993 to 2008, with a minimal postoperative followup of three years. One hundred and seventy-eight (87.3%), twenty (9.8%), and five (2.5%) of them were classified as basic type, simulated divergence excess type, and true divergence excess type intermittent exotropia, respectively, according to the Burian classification [9]. One hundred and fourteen of them were male and eighty-nine were female. The postoperative follow-up period ranged from a minimum of 3 years to a maximum of 8 years. The age of onset was 35.7 ± 26.9 months and the age at surgery was 96.5 ± 43.8 months. The interval between onset and surgery was 60.9 ± 37.4 months. The preoperative distance deviation was 31.0 ± 9.3 PD . The refractive error was −1.32 ± 2.30 D and anisometropia was 0.83 ± 1.33 D. Preoperatively, thirty-two patients had constant phase of intermittent exotropia and one hundred and seventy-one patients had intermittent phase. At 6 weeks postoperatively, 118 (58.1%) patients had successful outcome and 85 (41.9%) had unsuccessful outcome. At 3 years postoperatively, 97 (47.8%) had successful outcome and 106 (52.2%) had unsuccessful outcome.

### 3.1. Postoperative Drift Pattern over 3 Years

The postoperative drift pattern of our cohort is listed in Table 1. The postoperative drift tends to be towards exodrift. The proportion of exodrift increased from 62% at 6 weeks to 84% at 3 years postoperatively. The postoperative drift was 4.3 ± 8.1 PD at 6 weeks, 5.8 ± 8.4 PD at 6 months, 7.2 ± 8.3 PD at 1 year, 7.4 ± 8.4 PD at 2 years, and 7.7 ± 8.5 PD at 3 years.

### 3.2. Correlation Analysis of Factors Associated with Postoperative Drift at 6 Weeks and 3 Years

Table 2 revealed the factors associated with postoperative drift at 6 weeks and at 3 years. Preoperative deviation and initial overcorrection are correlated with postoperative drift both at 6 weeks and at 3 years. That is, a larger preoperative deviation is associated with a larger early and late postoperative drift. A larger initial overcorrection is also associated with a larger early and late postoperative drift.

### 3.3. Exodrift in Initial Overcorrection Group versus Initial Undercorrection Group

Patients were divided into initial overcorrection group (orthophoria to overcorrection) and initial undercorrection group, with 72 and 131 patients, respectively. The exodrift in the initial overcorrection group was significantly greater than that in initial undercorrection group at 6 weeks (8.2 ± 8.48 and 2.14 ± 7.03, resp., *P* < 0.001) and 3 years postoperatively (10.89 ± 9.83 versus 5.66 ± 7.33, resp., *P* < 0.001).

### 3.4. Exodrift in Group with Presence of Preoperative Stereopsis versus Group with Absence of Preoperative Stereopsis

Patients were divided into groups with presence and absence of preoperative stereopsis, with 174 and 29 patients, respectively. The exodrift in the group with presence of stereopsis was not significantly different from those with absence of stereopsis at 6 weeks (4.07 ± 8.33 and 5.59 ± 6.44, resp., *P* = 0.353) and at 3 years postoperatively (7.61 ± 8.68 and 8.03 ± 7.78, resp., *P* = 0.807).

### 3.5. Exodrift in Different Subtypes of Intermittent Exotropia

The exodrift in intermittent exotropia of basic type, simulated divergence excess type, and true divergence excess type was similar at 6 weeks (4.6 ± 7.5, 2.85 ± 12.5, and −1.2 ± 3.90, resp., *P* = 0.082 Kruskal Wallis test) and at 3 years postoperatively (7.8 ± 8.20, 8.2 ± 11.75, and 1.6 ± 3.0, resp., *P* = 0.078 Kruskal Wallis test).

### 3.6. Relationship between Postoperative Drift and Surgical Outcome

The mean postoperative drift between the success group versus unsuccessful group at different postoperative time points is listed in Table 3. From postoperative one year onwards, the postoperative drift in the successful group was significantly smaller than that in the unsuccessful group.

## 4. Discussion

We reported the postoperative drift pattern in our cohort of 203 patients with long term followup of at least 3 years. Consistent with previous studies, we demonstrated that majority of the patients demonstrated exodrift and, along 3-year period, the proportion of patients having the exodrift and the magnitude of the exodrift tended to increase with time. This is consistent with another study [10].

Many factors such as magnitude of preoperative deviation, distance-near disparity, age at surgery, refractive error, and type of surgery have been reported as predictive of outcome [11–15]. However only few studies have evaluated postoperative exodrift as a factor of the outcome [16]. Our results revealed that the surgical outcome is significantly affected by the exodrift. The exodrift in the success group is smaller than that in the failure group from 6 months onwards and the difference became statistically significant after 1 year onwards.

Consistent with Park and Kim we demonstrated that preoperative deviation and initial postoperative overcorrection were the most important factors correlating to the amount of postoperative drift [16]. That is, patients with larger preoperative deviations tend to have a larger postoperative drift. This may be the reason why preoperative deviation was found to be a crucial factor affecting the long term outcome. However, why a larger preoperative deviation would lead to larger postoperative drift remained unclear. Umazume et al. believed that the anatomical changes that normally take place in the muscle and fasciae are more extensive in those with larger preoperative deviations than those with smaller preoperative deviations and this difference may be related to the differences in surgical outcome [8].

A larger initial overcorrection is the second factor correlating with a larger postoperative exodrift. This is also observed by Ruttum [5], who reported a tendency towards a more postoperative exodrift with larger initial overcorrection. However, the mechanism underlying this observation required further investigations and explanations.

The study was limited by its retrospective nature. Nevertheless, our cohort has a relatively large sample size and long term followup which allow determination of the long term ocular stability of intermittent exotropia after bilateral lateral rectus recession.

## 5. Conclusions

Postoperative exodrift along three years occurs in majority of patients after bilateral lateral rectus recession for intermittent exotropia. The long term surgical success is significantly affected by this postoperative exodrift. A larger preoperative deviation and a larger initial overcorrection are associated with a larger early and late postoperative exodrift.

## Figures and Tables

**Table 1:** Pattern of postoperative drift of our 203 patients.

Postoperative time point	Number of esodrifts (%)	Number of no drift (%)	Number of exodrift (%)	Mean postoperative drift
6 weeks	30 (14.8)	47 (23.2)	126 (62.1)	4.3 ± 8.1 PD
6 months	26 (12.8)	27 (13.3)	150 (73.9)	5.8 ± 8.4 PD
1 year	23 (11.3)	17 (8.4)	163 (80.3)	7.2 ± 8.3 PD
2 years	23 (11.3)	14 (6.9)	166 (81.8)	7.4 ± 8.4 PD
3 years	21 (10.3)	11 (5.4)	171 (84.2)	7.7 ± 8.5 PD

**Table 2:** Correlation analysis to determine the influence of each preoperative parameter on postoperative drift at 6 weeks and 3 years.

Preoperative parameters	6 weeks		3 years	
	Correlation coefficient	*P* value	Correlation coefficient	*P* value
Age at onset	−0.14	0.05	−0.94	0.180
Age at surgery	−0.117	0.096	−0.130	0.064
Interval between onset and surgery	−0.034	0.627	−0.84	0.234
Preoperative deviation (PD) at distance	0.193	0.006	0.177	0.011
Refractive error (D)	0.041	0.564	0.113	0.108
Anisometropia (D)	−0.048	0.499	−0.121	0.085
Visual acuity RE (LogMAR)	−0.066	0.349	−0.004	0.950
Visual acuity LE (LogMAR)	−0.063	0.373	0.000	0.990
Phase of exotropia	−0.006	0.938	0.042	0.555
A- or V-pattern	−0.100	0.156	−0.121	0.085
Near-distance disparity	−0.058	0.408	−0.092	0.193
Presence of stereopsis	0.065	0.368	0.015	0.835
Type of horizontal surgery	0.005	0.940	0.101	0.151
Additional vertical surgery	−0.034	0.631	−0.031	0.658
Initial overcorrection angle	−0.472	0.000	−0.349	0.000

**Table 3:** Relationship between postoperative drift and surgical outcome.

Postoperative interval	Exodrift in success group	Exodrift in failure group	*P* value
6 weeks	4.5 ± 6.5	4.1 ± 9.9	0.743
6 months	5.1 ± 7.2	6.5 ± 9.6	0.237
1 year	5.4 ± 7.5	8.8 ± 8.8	0.003
2 years	5.4 ± 7.5	9.2 ± 8.9	0.001
3 years	5.2 ± 7.4	9.7 ± 9.2	<0.001
